# Calreticulin as A Novel Potential Metastasis-Associated Protein in Myxoid Liposarcoma, as Revealed by Two-Dimensional Difference Gel Electrophoresis

**DOI:** 10.3390/proteomes7020013

**Published:** 2019-04-10

**Authors:** Takashi Tajima, Fusako Kito, Akihiko Yoshida, Akira Kawai, Tadashi Kondo

**Affiliations:** 1 Division of Rare Cancer Research, National Cancer Center Research Institute, 5-1-1 Tsukiji, Chuo-ku, Tokyo 104-0045, Japan; t-tajima@ks.kyorin-u.ac.jp; 2 Department of Orthopaedic Surgery, Kyorin University School of Medicine, 6-20-2 Shinkawa, Mitaka, Tokyo 181-8611, Japan; 3 Department of Innovative Seeds Evaluation, National Cancer Center Research Institute, 5-1-1 Tsukiji, Chuo-ku, Tokyo 104-0045, Japan; fkitou@ncc.go.jp; 4 Pathology and Clinical Laboratory Division, National Cancer Center Hospital, 5-1-1 Tsukiji, Chuo-ku, Tokyo 104-0045, Japan; akyoshid@ncc.go.jp; 5 Division of Musculoskeletal Oncology, National Cancer Center Hospital, 5-1-1 Tsukiji, Chuo-ku, Tokyo 104-0045, Japan; akawai@ncc.go.jp

**Keywords:** myxoid liposarcoma, biomarker, metastasis, calreticulin, two-dimensional difference gel electrophoresis, mass spectrometry

## Abstract

Myxoid liposarcoma (MLS) is a mesenchymal malignancy. To identify innovate seeds for clinical applications, we examined the proteomes of primary tumor tissues from 10 patients with MLS with different statuses of postoperative metastasis. The protein expression profiles of tumor tissues were created, and proteins with differential expression associated with postoperative metastasis were identified by two-dimensional difference gel electrophoresis (2D-DIGE) and mass spectrometry. The validation was performed using specific antibodies and in vitro analyses. Using 2D-DIGE, we observed 1726 protein species and identified proteins with unique expression levels in metastatic MLS. We focused on the overexpression of calreticulin in metastatic MLS. The higher expression of calreticulin was confirmed by Western blotting, and gene silencing assays demonstrated that reduced expression of calreticulin inhibited cell growth and invasion. Our findings suggested the important roles of calreticulin in MLS metastasis and supported its potential utility as a prognostic biomarker in MLS. Further investigations of the functional properties of calreticulin and other proteins identified in this study will improve our understanding of the biology of MLS and facilitate novel clinical applications.

## 1. Introduction

Myxoid liposarcoma (MLS) is a common type of soft-tissue sarcoma, accounting for approximately one-third of all liposarcomas and 10% of all adult soft-tissue sarcomas [1]. Clinical features of MLS are diverse, ranging from localized and curable tumors to metastatic tumors, which can cause tumor-related death. MLS has unique molecular characteristics, including the recurrent translocation t(12:16)(q13:p11) and the corresponding chimeric protein FUS-CHOP (DDIT3, CREBP-homologous protein/DNA damage inducible transcript 3), which is a target of the drug trabectedin [2,3,4,5]. Despite the fact that the unique molecular features are well known in MLS, the origin of MLS remains obscure. Although fusion oncoproteins have critical roles in tumor formation, tumor progression is also correlated with round cell transformation [6]. Indeed, the presence of round cells is significantly associated with poor prognosis, and the 5-year survival rates in patients with round cells vary between 20% and 60%, depending on the round-cell histology. Several studies have demonstrated the aberrant activation of mammalian target of rapamycin, phosphatidylinositol 3-kinase, or phosphatase and tensin homolog pathways in round-cell variants [7,8,9]. However, our understanding of the mechanisms of metastasis and poor prognosis in patients with MLS is still limited, and further investigations are required to improve clinical outcomes in these patients.

Biomarkers are critical tools for optimizing therapy, and biomarker discovery has been extensively conducted from the early stage of proteomics [10]. Because cancer is a disease of the genome, and the proteome is a functional translation of the genome and directly determines cancer phenotypes, proteomics should be an ideal approach to cancer biomarker development. Unfortunately, there is no biomarker, which was discovered by proteomics and whose clinical utilities were proven in a clinical setting [11]. Thus, more efforts are needed to develop the novel proteomics modalities with higher technical performance and improve the experiment design using a higher number of samples. At the same time, as only a limited number of cancers were subjected to the proteomics study, we also need to challenge the non-examined cancers using the presently available methods.

Accordingly, in order to discover the biomarkers to predict the metastasis in patients with MLS, we examined the proteomic profiles of primary tumor tissues from MLS patients with different metastatic statuses. Proteome of tumor tissues of MLS has not been examined in association with clinical data until this paper. We created quantitative protein expression profiles and detected proteins associated with metastasis. Our results showed that many proteins were significantly associated with metastasis. Among them, we verified the biological significance of aberrant regulation of one of these proteins, calreticulin, using gene silencing assays, revealing its critical role in cell proliferation and migration.

## 2. Materials and Methods

### 2.1. Patients

This study included 10 patients with MLS who underwent curative resection from 1996 to 2007 at National Cancer Center Hospital (Tokyo, Japan). The patients in this study had not received neoadjuvant therapy at the time when the tumor tissue was sampled. The clinical and pathological characteristics of the patients in this study are summarized in Table 1. This study was approved by the ethical committees of National Cancer Center Hospital (2004-050), and the informed consent was obtained from all patients (Supplementary Materials).

### 2.2. Protein Expression Profiling

Proteins were extracted from frozen tumor tissues according to our previous report [12]. Briefly, tumor tissues were frozen after the surgical operation and stored until use at −80 degree. The frozen tissues were crushed and powdered in the presence of liquid nitrogen using a Multi-beads shocker (Yasui-kikai, Osaka, Japan). The tissues were then treated with protein lysis buffer (2 M thiourea, 6 M urea, 3% CHAPS, and 1% Triton X-100) for 30 min. Protease inhibitors were not used at any stage. After centrifugation at 15,000 rpm for 30 min, the supernatant was recovered and used as the protein lysate, and the resultant pellet was discarded.

Protein expression profiles were created using two-dimensional difference gel electrophoresis (2D-DIGE) according to our previous report [12]. Briefly, an internal standard sample was created by combining equal amounts of all individual protein samples. The internal standard sample was labeled with Cy3, individual samples were labeled with Cy5 (CyDye DIGE Fluor saturation dye; GE Healthcare, Uppsala, Sweden), and 5 μg of each labeled sample was combined and separated by two-dimensional polyacrylamide gel electrophoresis (2D-PAGE). Separation in the first dimension was achieved using a non-linear IPG dry-strip gel (GE Healthcare, Immobilize DryStrip, pH3-10 NL, 24 cm, GE Healthcare). The second dimension separation was achieved by SDS-PAGE using our original, large format gels (separation distance: 33 cm) [12]. The gels were scanned with laser scanners using appropriate wavelengths (Typhoon Trio; GE Healthcare). To compensate for gel-to-gel variations, Cy5 intensity was standardized against the intensity of Cy3 for all protein spots in an identical gel using image analysis software (Progenesis SameSpot; Nonlinear Dynamics, New Castle, UK). All samples were examined in triplicate, and the averaged standardized spot intensities were used for comparative studies. Proteome data were statistically examined using Expressionist software (Genedata, Basel, Switzerland).

### 2.3. Protein Identification

Proteins in the protein spots of interest were identified by mass spectrometry according to our previous report [12]. Briefly, 100 μg protein was labeled with Cy3 fluorescent dye (CyDye DIGE Fluor saturation dye; GE Healthcare) and separated by 2D-PAGE as described above. The proteins included in the protein spots were recovered as gel plugs using our original automated spot recovery machine and digested with modified trypsin (Promega, Madison, WI, USA). The trypsin digests were separated using liquid chromatography (Paradigm MS4 dual solvent delivery system; Michrom BioResources, Auburn, CA, USA) and subjected to LTQ linear ion trap mass spectrometry or LTQ Orbitrap XL analysis (ThermoElectron, San Jose, CA, USA) equipped with a nano-electrospray ion source (AMR, Tokyo, Japan). Mascot software (version 2.3.01; Matrix Science, London, UK) was used to search for peptide mass ion peaks against the SWISS-PROT database (*Homo sapiens*, 471,472 sequences in sprot_57.5 fasta file). Proteins with a Mascot ion score of at least 34 were used for protein identification.

### 2.4. Western Blotting

For separation by molecular weight, protein samples (5 μg) were separated by SDS-PAGE using 10% polyacrylamide gels (ATTO, Tokyo, Japan). For separation according to the isoelectric focusing point and molecular weight, protein samples (50 μg) prepared for 2D Western blotting experiments were separated using IPG dry-strip gels (pI range: 3–10; length: 18 cm; GE Healthcare) for the first dimension and 10% SDS-PAGE (ATTO) for the second dimension. 2D separation was performed as mentioned above. Following electrophoresis, proteins were transferred to polyvinylidene difluoride membranes (Immun-Blot PVDF Membrane for Protein Blotting, Bio-Rad, Hercules, CA) and reacted with anti-calreticulin antibodies (rabbit polyclonal antibody, anti-Calreticulin (#06-661) Upstate, Merck Millipore, Burlington, MA) at a dilution of 1:1000. After incubation with secondary antibodies (ECL Anti-rabbit IgG, Horseradish Peroxidase-Linked Species-Specific F(ab’)2 Fragment, GE Healthcare) at a dilution of 1:2000, immunoreactive spots were detected by enhanced chemiluminescence (ECL PRIME; GE Healthcare) and LAS-3000 (FujiFilm, Tokyo, Japan). The intensity of each protein spot was quantified (ImageQuant; GE Healthcare).

### 2.5. Immunohistochemistry

Formalin-fixed paraffin-embedded tissues were sectioned and subjected to immunohistochemical analysis. The sectioned tissues were reacted with antibodies against calreticulin (mouse monoclonal antibody; FMC 75; cat. no. ab22683; Abcam, Cambridge, UK) at a dilution of 1:1000 or Negative Control Mouse IgG1 (cat. no. X0931; Dako) at a dilution of 1:4000. After blocking with skim milk, the sectioned tissues were reacted with EnVision+System-HRP Labelled Polymer Anti-mouse Antibodies (cat. no. K4001; Dako).

### 2.6. Gene Silencing Assay

2645/94 MLS cells were previously established elsewhere [13] and kindly provided by Professor Masahiko Kuroda (Department of Molecular Pathology, Tokyo Medical University, Tokyo, Japan). The cells were maintained using DMEM and 10% FBS. Small interfering RNA (siRNA) specific for calreticulin was purchased from Sigma-Aldrich (St. Louis, MO, USA); the target sequences were 5′-CAGUAUCUAUGCCUAUGAU-3′ (HS02-00337402: siRNA-1), 5′-GAGUAUUCGCCCGAUCCCA-3′ (HS01-00137572: siRNA-2), and 5′-GUAUUCUCCCGAUCGCAGU -3′ (HS02-00337403: siRNA-3). 2645/94 MLS cells were transfected with siRNAs or a negative control (AllStars Negative Control siRNA; Qiagen, Valencia, CA, USA) using Lipofectamine RNAiMAX reagent (Invitrogen) according to the manufacturer’s protocol.

### 2.7. Cell Proliferation Assay

2645/94 cells transfected with calreticulin siRNAs or negative control siRNA were seeded in 96-well plates at 5000 cells/well. Cell viability was measured using a Cell Counting Kit-8 (Dojindo, Kumamoto, Japan) according to the manufacturer’s instructions. After 2 h, the number of viable cells were determined by measuring the absorbance at 450 nm with a SAFIRE plate reader (TECAN, Männedorf, Switzerland). Cell proliferation was examined every 24–72 h after seeding.

### 2.8. Cell Invasion Assay

Cell invasion was evaluated using BD Biocoat Matrigel Invasion Chambers (BD Biosciences, Bedford, MA, USA). Briefly, 2645/94 cells were transfected with calreticulin siRNA-1, siRNA-2, or siRNA-3 or negative control siRNA. Cells were seeded into the upper chambers at 0.5 × 10^5^ cells/well in serum-free RPMI medium, and medium containing 10% fetal bovine serum was added to the lower chamber. Forty-eight hours later, after fixation and staining cells, the numbers of cells on the bottom surface were counted in three separates areas at 100× magnification, and ratios of the number of cells in the Matrigel chamber to the number of cells in the control chamber (% invasion) were calculated.

## 3. Results

### 3.1. Protein Expression Profiles of MLS Cells Using 2D-DIGE

**Figure 1:**
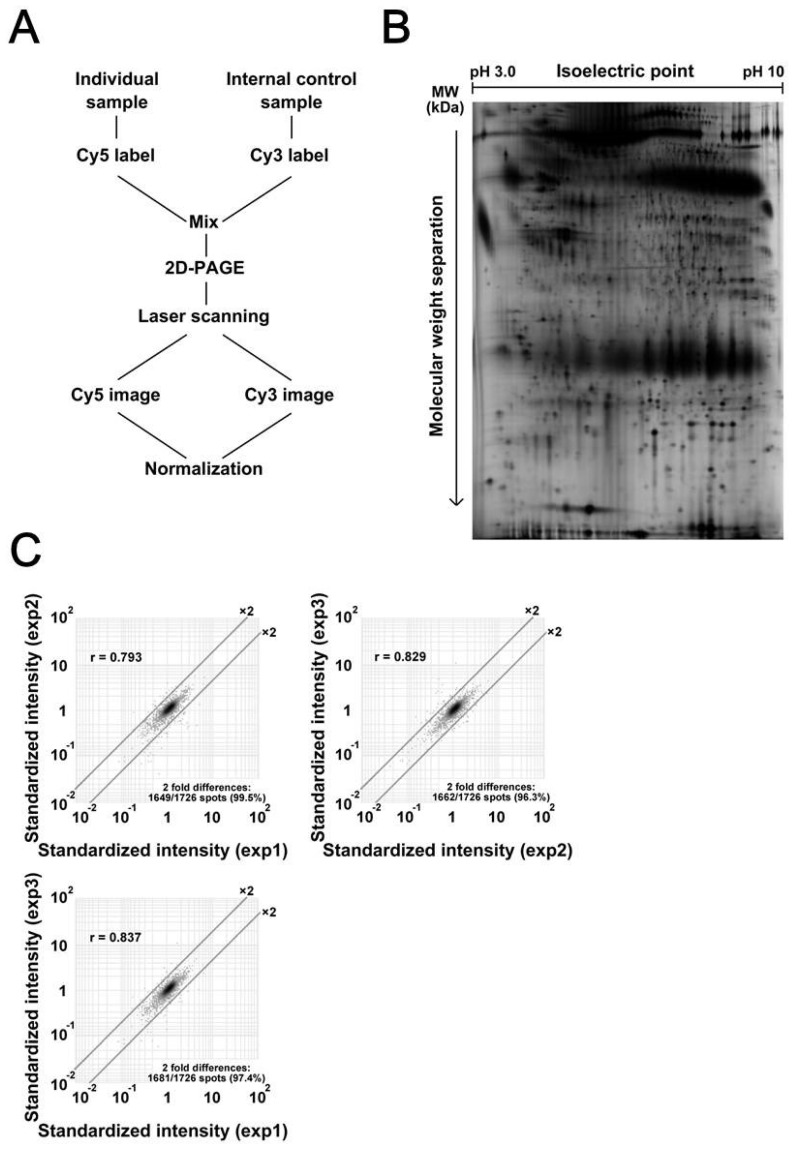
Overview of the proteomic study using two-dimensional difference gel electrophoresis (2D-DIGE). **(A**) The internal control sample and individual sample were labeled with Cy3 and Cy5, respectively, mixed together, and then separated by two-dimensional polyacrylamide gel electrophoresis (2D-PAGE). The gel was scanned using a laser scanner, and the Cy3 and Cy5 images were obtained. To normalize for gel-to-gel variations, the intensity of protein spots on the Cy5 image was normalized to the intensity of corresponding protein spots on the Cy3 image. (**B**) A typical Cy3 image is shown. Proteins were separated according to their isoelectric point and then their molecular weight. Note that proteins were well separated without remarkable streaking, and the image is not distorted overall. (**C**) The system repeatability of 2D-DIGE data was evaluated by running identical samples three times.

Protein expression profiles of primary tumor tissues were created by 2D-DIGE. Figure 1A illustrates the design of experiments using an internal standard sample (Figure 1A). All gels included the Cy3 image of common internal standard sample. By standardizing the Cy5 intensity of individual protein samples with the Cy3 intensity of corresponding protein spots of an internal standard sample, we compensated for gel-to-gel variations. Using a large-format gel electrophoresis apparatus, we observed 1726 protein species in single 2D-gel images. A typical proteome image is shown in Figure 1B, and its enlarged image is shown in Supplementary Figure S1. Note that all protein spots were well focused and separated from each other in all regions of the gel images. System repeatability was verified by scatter grams using data from triplicate experiments with identical samples (Figure 1C). We found that the standardized intensities of at least 96.3% of protein spots scattered within two-fold differences, with a correlation value of more than 0.793. According to the results of scatter gram analysis, we determined that when the spot intensity was different more than two times, that difference is significant in this experiment system.

### 3.2. Comparative Study between Sample Groups with Different Metastatic Statuses

**Figure 2:**
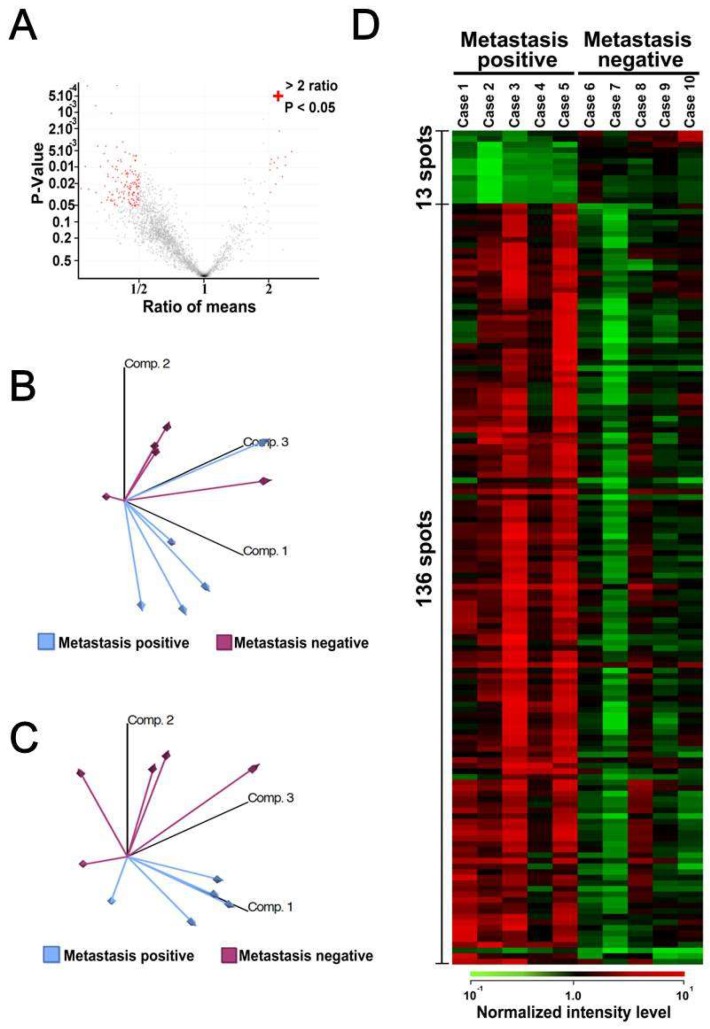
Overall differences between metastatic and nonmetastatic tumor samples. (**A**) The average intensity of protein spots was compared between metastatic and nonmetastatic samples. Volcano plots show protein spots with more than two-fold differences with statistical significance (*p* < 0.05). The 149 protein spots are marked with red. (**B**) Principal component analysis using all 1726 protein spots, showing the overall protein expression patterns according to the status of metastasis. (**C**) Principal component analysis using the 149 protein spots, showing separation between metastasis-positive and metastasis-negative samples. (**D**) Identification of protein spots, summarized in a heat-map.

We compare the proteome data obtained by 2D-DIGE between the patients with metastasis (cases 1–5, Table 1) and those without metastasis (cases 6–10, Table 1). The patients who had metastasis after surgical operation received chemotherapy (cases 1–5). One of those five patients had died when we performed this study. In contrast, the patients who did not have metastasis (cases 6–10) did not receive adjuvant chemotherapy, and all of them were alive when this study was conducted. We compared the spot intensity between these two patient groups and identified the protein spots with differential intensity with statistical significance. The proteins were extracted from the identified protein spots and subjected to mass spectrometry for protein identification. The distribution of protein spot intensities is shown as a function of *p* values and fold differences between samples from patients with or without metastasis (Figure 2A). The intensities of 149 protein spots were significantly different between the two sample groups (Figure 2A).

Unsupervised classification by principal component analysis using all proteome data suggested that the protein samples could be classified according to the metastatic status. Moreover, when the intensities of the selected 149 protein spots were used, the sample groups were further separated (Figure 2C). These observations suggested that the overall features of the proteome may be associated with the metastatic status and that the selected proteins may represent the characteristics of two sample groups. The intensities of the 149 protein spots in the 10 samples are illustrated as a heat-map (Figure 2D), and its enlarged image is shown in Supplementary Figure S2. The spot intensities seemed to be homogenous in each sample group, and the intensity difference was obvious between the sample groups. We found that the intensities of 13 and 136 protein spots were higher and lower, respectively, in tumors with metastasis. Among the 149 protein spots with different intensities, proteins corresponding to the 148 protein spots were identified by mass spectrometry.

The results of comparative analyses and protein identification are summarized in Table 2. The protein spots listed in Table 2 had significantly different intensities between metatstatic and non-metastatic MLS.

### 3.3. Calreticulin Expression was Associated with Metastasis

**Figure 3:**
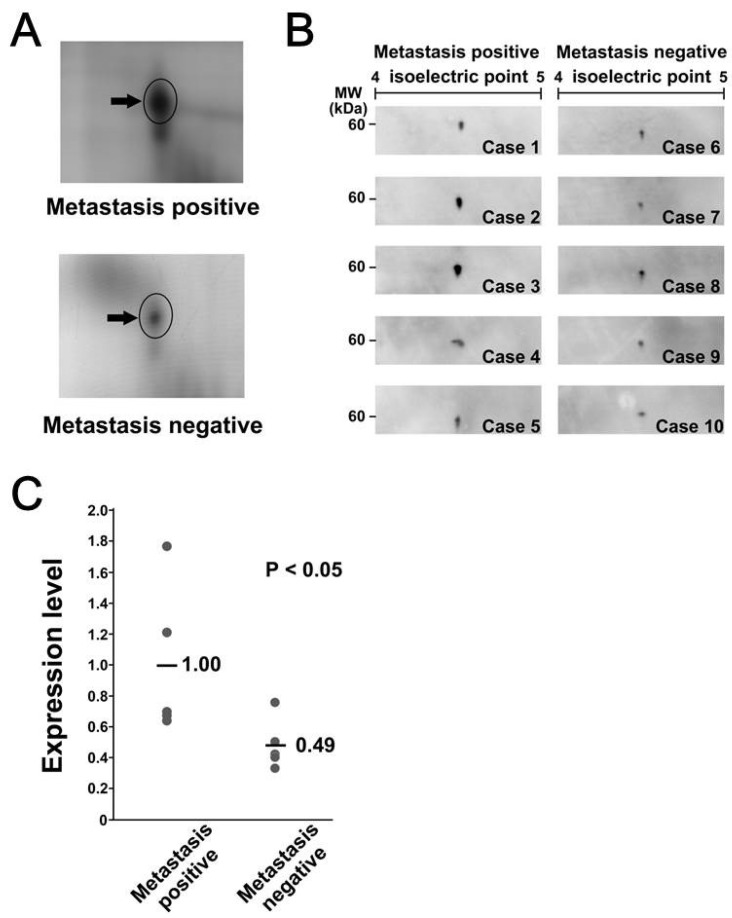
Calreticulin was overexpressed in metastasis-positive samples. (**A**) Close-up image of the protein spot of calreticulin in metastasis-positive and metastasis-negative samples. (**B**) Western blotting confirmed the differential expression of calreticulin. The specific antibody was reacted with the membrane to which two-dimensionally separated protein samples were transferred. (**C**) The intensity of protein spots on Western blotting was quantified and summarized in the graph.

We further focused on the overexpression of calreticulin in MLS. We validated the overexpression of calreticluin in MLS with metastasis by Western blotting. First, we performed SDS-PAGE/Western blotting. However, we did not detect significant differences in intensity associated with metastasis (data not shown), probably because of possible posttranslational modifications or the presence of isoforms without expression difference. Alternatively, the proteins which have an epitope reacting to the anti-calreticulin antibody may exist at the same location of calreticulin in the SDS-PAGE gel. We then performed 2D-PAGE/Western blotting. In 2D-PAGE/Western blotting, calreticulin was observed as a single protein spot, suggesting that there were no obvious post-translational modifications (Figure 3A). Quantification of Western blotting indicated that the expression level of calreticulin in 2D-PAGE/Western blotting was significantly higher in tumor tissues with metastasis than in those without metastasis (*p* < 0.05), with average differences of more than two-fold (Figure 3B and C). These observations were consistent with the results of 2D-DIGE.

### 3.4. Immunohistochemical Localization of Calreticulin in Tumor Cells

We localized calreticulin in tumor tissues using immunohistochemistry. Using sectioned tumor tissues from all nine patients with MLS in this study, we found that there were no significant differences in calreticulin expression (Figure S3).

### 3.5. In Vitro Functional Analysis of Calreticulin

**Figure 4:**
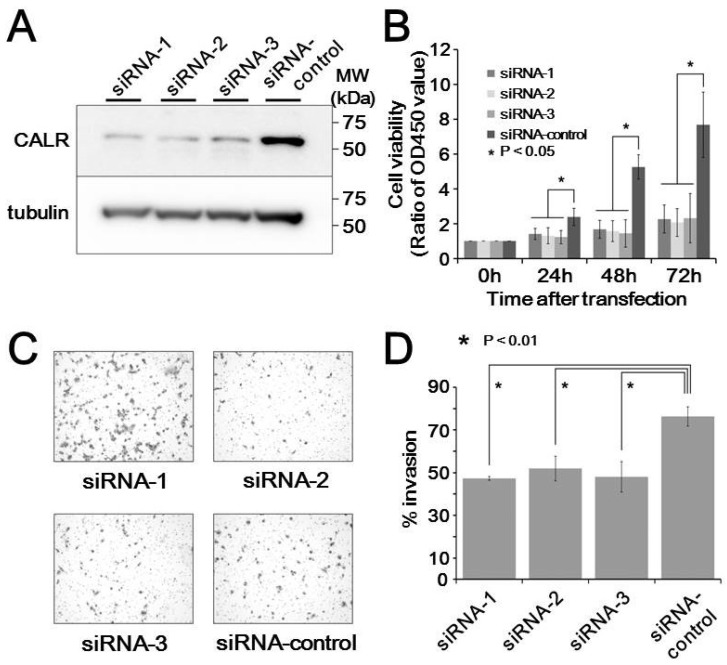
Effects of calreticulin on cell behaviors in myxoliposarcoma cells. (**A**) Expression of calreticulin in response to transfection with three siRNAs and a control siRNA. (**B**) Viability of transfected cells compared with that in control cells. (**C**) Transwell invasion assays in siRNA-transfected cells. (**D**) Quantification of the data from (**C**).

The functional significance of calreticluin upregulation in metastatic MLS tissues was examined in cultured MLS cells. Transfection of 2645/94 cells with siRNAs against calreticulin resulted in considerable reduction of calreticulin expression compared with that in cells treated with control siRNA (Figure 4A). Cell growth assays showed that siRNA silencing of calreticulin resulted in decreased cell proliferation compared with that in control siRNA-transfected cells (*p* < 0.05, Figure 4B). Moreover, siRNA-mediated silencing of calreticulin significantly upregulated the invasiveness of MLS cells (*p* < 0.01, Figure 4C,D). These observations suggest that calreticulin promoted tumor progression in MLS cells.

## 4. Discussion

Profiling of the genes relevant to metastasis is the first step to understanding the mechanisms of malignant behaviors in tumors. Although the cancer biomarker discovery has been performed in many cancers using proteomics, there was no report about metastasis biomarkers of MLS until our study. Here, using a set of tumor tissue samples from the patients with different metastasis status, we examined the proteome and identified differentially expressed proteins. Those proteins are potentially biomarker candidates, and we focused on one of the identified proteins, calreticulin.

Calreticulin is a Ca^2+^-binding chaperon protein predominantly located in the endoplasmic reticulum [14]. Higher levels of calreticulin have been reported in various types of cancers [15] and are associated with clinical stages and metastasis in breast cancer [16], gastric cancer [17], prostate cancer [18], and ovarian cancer [19]. Moreover, the overexpression of calreticulin is relevant to shorter survival in pancreatic cancer [20] and esophageal cancer [21]. In bladder urothelial cancer, calreticulin in urine is a candidate biomarker [22]. These reports have suggested that calreticulin plays important roles in carcinogenesis and cancer progression. The possible mechanisms through which calreticulin mediates metastasis have been demonstrated in previous reports. The interaction of calreticulin with integrin *α*2*β*1 was observed in cells with active integrin [23], and calreticulin regulates integrin activation by modifying the *α*1,2-linkaged glycomic status of *β*1-integrin [24]. Calreticuln affects cell adhesion and migration by altering the components of the extracellular matrix, including fibronectin [25]. Proteins involved in the regulation of focal contacts by calreticulin include the calmodulin/calmodulin-dependent kinase II pathway [26] and vinculin [27]. Calreticulin also functions as an angiogenesis factor. Calreticulin positively regulates vascularization through the activation of vascular endothelial growth factor (VEGF) in gastric cancer [17]. Interestingly, vasostatin, which is a fragment of calreticulin, is considered an anti-angiogenic factor and inhibits VEGF-induced enthothelial cell proliferation [28]. These reports suggested that various mechanisms may explain the association of calreticulin with metastasis in various cancers.

Aberrant expression of carleticulin has been reported in sarcomas. For example, Hisaoka et al. investigated the expression of calreticulin in lipogenic tumors and other sarcomas [29]. They found that calreticulin was frequently overexpressed in dedifferentiated areas of dedifferentiated liposarcomas compared with that in normal surrounding tissue. Moreover, they demonstrated the various expression levels of calreticulin in MLS cases, without describing the association of calreticulin with metastasis. In our study, differences in calraticulin levels between metastatic and nonmetastatic MLS were detected by 2D-DIGE and 2D/Western blotting, but not by immunohistochemistry. The differences in methods may cause the apparent discrepancy. Indeed, protein detection can often vary depending on the method used. 2D-DIGE using saturation dyes detects proteins with cysteine residues after the proteins are highly separated by two-dimensional gel electrophoresis. Thus, proteins that are not labeled by fluorescent dyes, not separated well by electrophoresis, or not expressed at sufficiently high levels may not be identified by 2D-DIGE. In SDS-PAGE/Western blotting, the proteins are heated, separated according to their molecular weight, and transferred to membranes. Proteins that do not exhibit antigenic sequences on the membranes will not be detected. In 2D/Western blotting, proteins are separated by two-dimensional gel electrophoresis and transferred to membranes. Moreover, proteins showing antigenic sequences under these conditions may be detected by antibodies. In immunohistochemistry, the proteins are fixed with formalin and embedded in paraffin. After the fixed tissues are sliced, the proteins are detected by antibodies. Thus, proteins having sequences modified by formalin may react differently with antibodies. Western blotting is best suited for research in the laboratory because it is possible to obtain information regarding protein structure from electrophoretic separation. In contrast, immunohistochemical detection is best suited for confirming the localization of proteins and to perform multi-institutional validation studies.

We found that the knockdown of calreticulin inhibited proliferation and invasion in MLS cells. These observations were concordant with previous reports in other malignancies. In oral cancer cells, depletion of calreticulin was found to result in cell cycle arrest at the G_0_/G_1_ phase, suppressing growth rates, colony-formation capacity, and anchorage-independent growth [30]. Additionally, knockdown of calreticulin suppresses the growth, adhesion, and migratory ability of the cells and blocks in vivo metastasis in bladder cancer [31]. In this study, we firstly confirmed the possible contribution of calreticulin to the malignant characters of MLS. These observations suggest that cancer cells with different histological and genetic backgrounds may share some common mechanisms for proliferation, metastasis and invasion.

The limitation of this study is firstly the small number of cases. Further studies are needed to assess the future clinical applications of MLS overexpression. MLS is a rare malignancy, and the frozen tissues are not routinely stored in the hospitals. The results of this study should be validated in the newly enrolled cases, and for this sake, we need to conduct a multi-institutional study. Secondly, we need to elucidate the mechanisms underlying the correlation between higher expression of calreticulin and metastasis in MLS. Thirdly, although we demonstrated the utility of the proteomic approach using 2D-DIGE to find proteins associated with metastasis in MLS, the total number of proteins observed in this study was limited. The pros and cons of 2D-DIGE for biomarker discovery were previously discussed [32], and it may be worth challenging other proteomics modalities such as label-free mass spectrometry and protein arrays. The other protein extraction and fraction methods are worth considering too. The proteome data are always biased by the technical characters of proteomics modalities, and a perfect proteome map is not available. However, as long as the same protocol is used for the comparative study, the results are reliable. Finally, although there are many proteins identified in this study as metastasis-associated ones, we focused only on calreticulin and did not study the rest of the identified proteins. The proteins identified but not examined in this study are also worth investigating.

We found that the expression of calreticulin was significantly correlated with metastasis. If the correlation is established after an extensive validation study, we will be able to predict the metastasis using the surgically resected tumor tissue and optimize the therapeutic strategy.

## 5. Conclusions

In this study, we found an association between the higher expression of calreticulin and metastasis in MLS. Further studies are worth conducting to reveal the possible clinical applications of our findings.

## Figures and Tables

**Table 1:** Clinical backgrounds of the patients with myxoid liposarcomain this study.

Case No.	Gender	Age	Location	Metastasis	Metastasis Duration (month)		Therapy for Advanced Disease	Follow-Up Duration (month)	Follow-Up Status
1	female	51	thigh	positive	19	CTx ^1^ and RTx ^2^		21	DOD ^3^
2	female	61	thigh	positive	17	CTx and RTx		75	AWD ^4^
3	Male	31	forearm	positive	65	CTx		84	DOD
4	Male	69	buttock	positive	7	CTx and RTx		41	DOD
5	Male	41	foot	positive	0	CTx		21	DOD
6	Male	44	thigh	negative	-	-		169	CDF ^5^
7	female	52	inguinal	negative	-	-		150	CDF
8	female	35	lower leg	negative	-	-		88	CDF
9	Male	65	thigh	negative	-	-		77	CDF
10	female	47	thigh	negative	-	-		68	CDF

^1^ CTx: chemotherapy, ^2^ RTx: radiotherapy, ^3^ DOD: dead of disease, ^4^ AWD: alive with disease, ^5^ CDF: continuous disease free.

**Table 2:** Proteins with differential expression between tumor tissues with metastasis and those without metastasis.

Spots No.	Welch Test *p*-Value	Ratio of Means	Identified Protein
44	1.31E-02	0.42	Endoplasmin
62	4.78E-02	0.38	Keratin, type I cytoskeletal 10
74	3.15E-02	0.38	Keratin, type II cytoskeletal 1
85	9.56E-03	0.28	Trypsin-1
91	2.82E-02	0.36	Trypsin-1
163	4.96E-02	0.39	Keratin, type II cytoskeletal 1
177	4.58E-02	0.38	Keratin, type II cytoskeletal 2 epidermal
182	3.49E-02	0.36	Trypsin-1
185	1.46E-02	0.36	Aconitate hydratase, mitochondrial
188	2.02E-02	0.76	Ig mu chain C region
216	6.69E-03	2.19	Keratin, type II cytoskeletal 2 epidermal
221	9.04E-03	2.38	Serum albumin
222	8.09E-03	2.11	Serum albumin
223	1.11E-02	2.01	Cancer-associated gene 1 protein
228	2.84E-02	2.03	Serum albumin
229	1.46E-02	2.30	Serum albumin
230	6.48E-03	2.38	Serum albumin
232	8.57E-03	2.02	Serum albumin
276	7.57E-03	0.40	Keratin, type II cytoskeletal 1
277	4.07E-03	0.32	78 kDa glucose-regulated protein
279	4.09E-03	0.35	Trypsin-1
280	1.49E-02	0.49	Glutamine-rich protein 2
281	3.24E-04	0.29	78 kDa glucose-regulated protein
282	3.46E-03	0.42	Keratin, type I cytoskeletal 10
286	2.75E-02	0.42	Trypsin-1
340	2.29E-02	0.48	Calreticulin
344	3.01E-02	0.47	Uncharacterized protein KIAA1529
365	1.63E-02	0.50	Alpha-1-antichymotrypsin
397	2.92E-02	0.40	Very long-chain specific acyl-CoA dehydrogenase, mitochondrial
469	2.12E-02	0.43	Keratin, type II cytoskeletal 1
511	1.30E-02	0.44	Vimentin
559	2.28E-02	0.46	Keratin, type II cytoskeletal 1
562	1.68E-02	0.49	Trypsin-1
569	1.96E-02	0.46	6-phosphogluconate dehydrogenase, decarboxylating
590	1.48E-02	0.44	Keratin, type I cytoskeletal 10
598	1.82E-02	0.44	Keratin, type II cytoskeletal 1
647	2.21E-02	0.45	Keratin, type I cytoskeletal 10
680	4.46E-02	0.47	Keratin, type II cytoskeletal 1
694	2.21E-02	0.44	Keratin, type II cytoskeletal 1
702	4.46E-02	0.45	Fumarate hydratase, mitochondrial
713	3.92E-02	0.35	Serpin H1
742	2.25E-02	0.48	Trypsin-1
744	4.98E-02	0.47	Growth factor receptor-bound protein 1
762	3.23E-02	0.45	Keratin, type II cytoskeletal 1
787	4.99E-02	0.48	Protein Shroom3
788	2.14E-02	0.45	Glutamate receptor, ionotropic kainate 3
790	5.59E-03	0.34	Guanine nucleotide-binding protein G(I)/G(S)/G(T) subunit beta-1
802	4.20E-02	0.42	Keratin, type II cytoskeletal 1
809	2.25E-02	0.43	3-ketoacyl-CoA thiolase, mitochondrial
810	1.19E-02	0.46	Cytochrome b-c1 complex subunit 2, mitochondrial
811	3.14E-02	0.37	Trypsin-1
841	3.97E-02	0.47	Heterogeneous nuclear ribonucleoprotein D0
848	2.15E-02	0.49	Trypsin-2
864	6.12E-03	0.48	Guanine nucleotide-binding protein G(i), alpha-2 subunit
903	9.55E-03	0.46	Keratin, type I cytoskeletal 9
986	3.96E-02	0.50	Annexin A2
988	2.39E-02	0.45	Keratin, type I cytoskeletal 10
995	2.85E-02	0.47	Keratin, type I cytoskeletal 10
1000	4.91E-02	0.46	L-lactate dehydrogenase B chain
1007	3.03E-02	0.42	Keratin, type II cytoskeletal 1
1008	3.87E-02	0.37	Keratin, type II cytoskeletal 1
1015	4.07E-02	0.38	Keratin, type I cytoskeletal 10
1054	7.46E-04	0.31	Insulin receptor
1103	2.42E-02	0.27	Transmembrane protease, serine 13
1148	1.88E-02	0.48	Heterogeneous nuclear ribonucleoproteins A2/B1
1169	7.59E-03	0.36	Glutamine-rich protein 2
1216	1.34E-02	0.44	Probable ATP-dependent RNA helicase DDX56
1231	3.64E-02	0.50	Tropomyosin alpha-3 chain
1246	2.71E-02	0.39	Keratin, type II cytoskeletal 1
1390	5.25E-03	0.43	Zinc finger CCHC domain-containing protein 11
1397	4.35E-02	0.33	Keratin, type II cytoskeletal 2 epidermal
1401	4.83E-02	0.48	Keratin, type I cytoskeletal 10
1421	1.16E-02	0.48	Lethal(2) giant larvae protein homolog 2
1452	2.00E-02	0.48	Uncharacterized protein KIAA1529
1480	4.67E-02	0.46	Lethal(2) giant larvae protein homolog 2
1512	4.27E-02	0.48	Uncharacterized protein KIAA1529
1515	9.61E-03	0.33	CD83 antigen
1526	5.63E-03	0.45	Uncharacterized protein KIAA1529
1556	2.99E-02	0.45	Glutamine-rich protein 2
1593	2.46E-02	0.45	Zinc finger protein 616
1599	1.08E-02	0.39	Keratin, type II cytoskeletal 1
1606	9.59E-03	0.48	ADP-ribosylation factor 1
1607	2.00E-02	0.37	Peptidyl-prolyl cis-trans isomerase B
1628	2.29E-02	0.45	Uncharacterized protein KIAA1529
1655	7.58E-03	0.48	LIM and calponin homology domains-containing protein 1
1677	3.13E-02	0.50	Echinoderm microtubule-associated protein-like 6
1700	3.14E-04	0.39	Keratin, type I cytoskeletal 10
2381	2.87E-02	0.31	Aconitate hydratase, mitochondrial
2382	1.02E-02	2.18	Uncharacterized protein KIAA1529
2396	7.10E-03	2.04	RNA-binding protein NOB1
2398	2.15E-03	2.23	Uncharacterized protein KIAA1529
2404	5.14E-03	2.54	Keratin, type I cytoskeletal 10
2409	2.52E-02	0.45	Teneurin-1
2413	3.00E-02	0.50	Moesin
2419	2.82E-02	0.42	Trypsin-2
2426	3.59E-02	0.42	Heat shock cognate 71 kDa protein
2435	1.51E-02	0.40	Growth arrest-specific protein 2
2512	3.66E-02	0.49	Glutamine-rich protein 2
2513	1.88E-02	0.46	Glutamine-rich protein 2
2534	3.22E-02	0.47	Keratin, type I cytoskeletal 10
2543	4.61E-02	0.46	Acetolactate synthase-like protein
2546	1.80E-02	0.39	Keratin, type I cytoskeletal 10
2548	1.13E-02	0.45	Keratin, type I cytoskeletal 10
2550	1.86E-02	0.47	Crumbs homolog 1
2551	7.32E-03	0.48	Keratin, type I cytoskeletal 10
2559	1.31E-02	0.47	Vimentin
2584	3.45E-02	0.41	Uncharacterized protein KIAA1529
2586	1.88E-02	0.41	Transcriptional adapter 3-like
2593	3.80E-02	0.47	Complement C2
2616	2.05E-02	0.43	Coiled-coil domain-containing protein 116
2633	3.94E-02	0.38	Serpin H1
2634	7.83E-03	0.41	Serpin H1
2637	7.27E-03	0.41	Keratin, type II cytoskeletal 2 epidermal
2639	2.95E-02	0.49	Zinc finger protein 512
2640	3.84E-02	0.49	Not identified
2643	3.58E-02	0.40	Serpin H1
2644	4.81E-02	0.50	Serpin H1
2652	2.00E-02	0.47	Glutamine-rich protein 2
2653	1.30E-02	0.43	Actin, cytoplasmic 1
2663	2.23E-02	0.50	Glutamine-rich protein 2
2667	4.24E-03	0.39	Keratin, type II cytoskeletal 1
2674	1.93E-02	0.50	Keratin, type I cytoskeletal 10
2692	1.38E-02	0.50	Actin, cytoplasmic 1
2695	4.04E-02	0.37	Mitogen-activated protein kinase kinase kinase 8
2699	1.11E-02	0.40	Actin, cytoplasmic 1
2700	9.58E-03	0.39	Actin, cytoplasmic 1
2701	2.18E-02	0.35	Keratin, type II cytoskeletal 1
2709	3.71E-02	0.43	Echinoderm microtubule-associated protein-like 6
2710	1.52E-02	0.38	Keratin, type I cytoskeletal 10
2783	2.46E-02	0.39	Stress-70 protein, mitochondrial
2791	3.75E-02	0.46	Uncharacterized protein KIAA1529
2792	1.02E-03	0.37	Uncharacterized protein KIAA1529
2796	1.72E-02	0.49	Poly(ADP-ribose) glycohydrolase ARH3
2812	1.24E-02	0.44	Transmembrane protein 18
2819	1.33E-02	0.36	Glutamine-rich protein 2
2834	3.37E-02	0.48	Zinc finger protein 584
2837	3.88E-02	0.45	Uncharacterized protein KIAA1529
2868	3.12E-02	0.46	Uncharacterized protein KIAA1529
2878	1.36E-02	0.48	Hydroxyacyl-coenzyme A dehydrogenase, mitochondrial
2929	2.47E-02	0.49	Growth factor receptor-bound protein 10
2931	6.12E-03	0.47	Apolipoprotein A-I
2934	4.76E-02	0.49	Apolipoprotein A-I
2951	2.17E-02	0.50	FAST kinase domain-containing protein 1
2959	1.16E-02	0.43	Uncharacterized protein KIAA1529
2964	4.21E-02	0.45	Kelch-like protein 38
2971	2.88E-02	0.47	Keratin, type II cytoskeletal 1
2985	2.49E-02	0.29	Keratin, type I cytoskeletal 10
2993	2.87E-02	0.47	Glutamine-rich protein 2
2997	6.08E-03	0.48	Keratin, type I cytoskeletal 10

## Supplementary Materials

The following are available online at https://www.mdpi.com/2227-7382/7/2/13/s1. Figure S1: Enlarged image of 2D-DIGE. Figure S2: Enlarged image of heat map in Figure 2D. Figure S3: Images of immunohistochemical analysis of calreticulin.
