# Supplementary figures and images for: Calreticulin as A Novel Potential Metastasis-Associated Protein in Myxoid Liposarcoma, as Revealed by Two-Dimensional Difference Gel Electrophoresis
Source: Proteomes. 2019 Apr 10;7(2):13. doi: 10.3390/proteomes7020013 (PMC6631384; doi:10.3390/proteomes7020013)

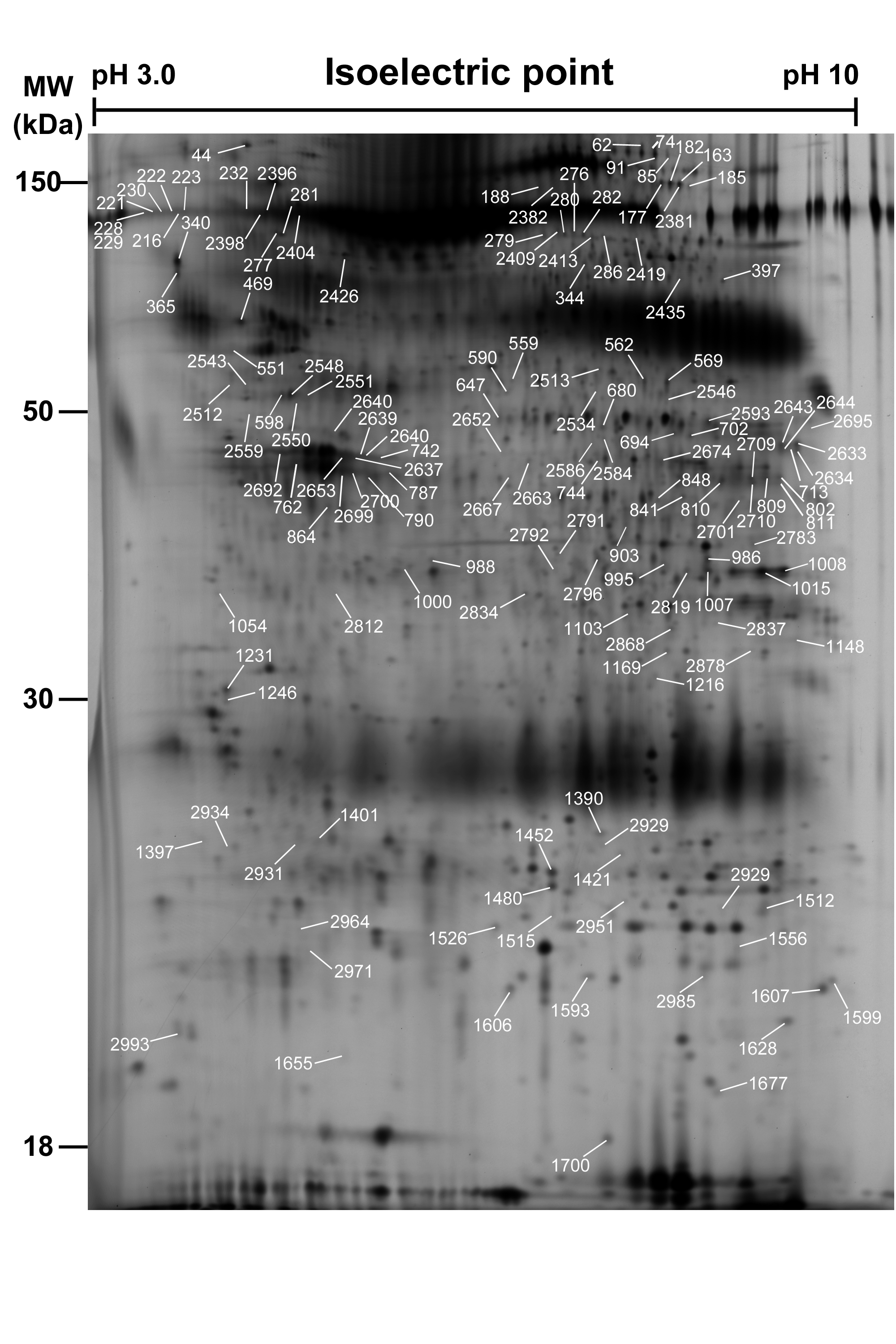

Supplement: Supplementary file 1 [file proteomes-07-00013-s001.zip › proteomes-452534 supplementary for final/Supplementary Figure S1.tif]

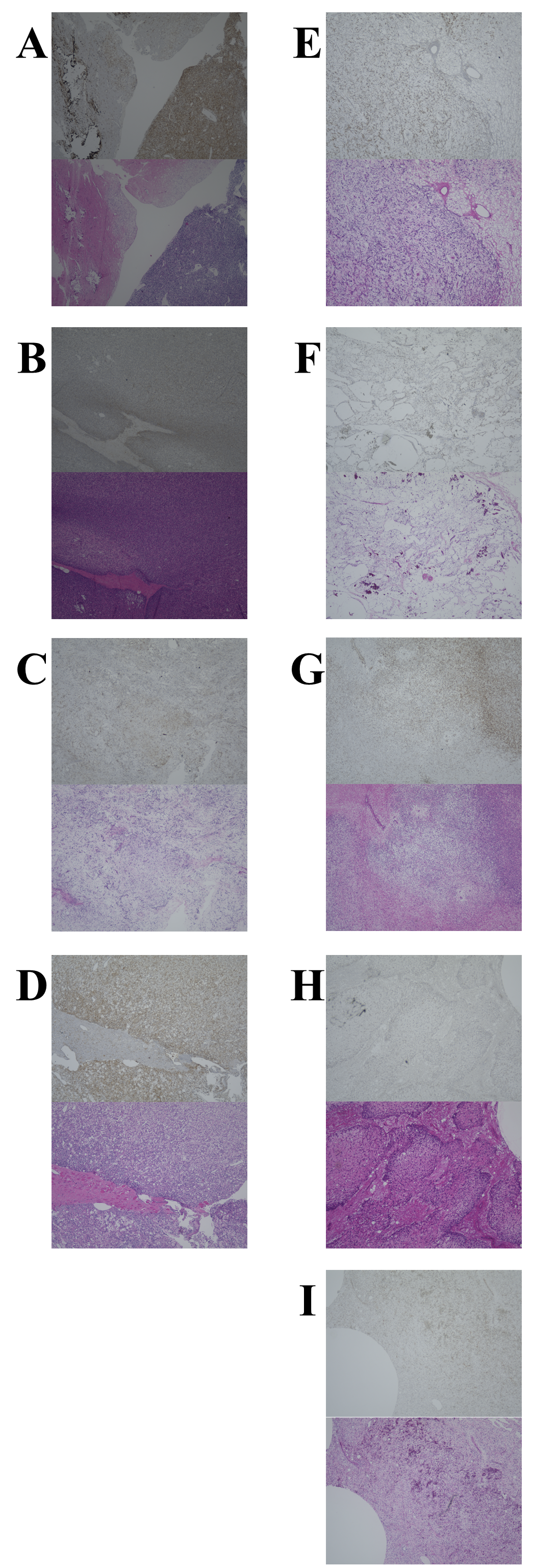

Supplement: Supplementary file 1 [file proteomes-07-00013-s001.zip › proteomes-452534 supplementary for final/Supplementary Figure S3.tif]
